# The impacts of New York's balance billing regulation on ground ambulance pricing

**DOI:** 10.1111/1475-6773.14387

**Published:** 2024-10-16

**Authors:** Wendy Y. Xu, Christopher Garmon, Sheldon M. Retchin, Yiting Li

**Affiliations:** ^1^ Division of Health Services Management and Policy, College of Public Health The Ohio State University Columbus Ohio USA; ^2^ Henry W. Bloch School of Management The University of Missouri – Kansas City Kansas City Missouri USA; ^3^ Division of General Internal Medicine, College of Medicine The Ohio State University Columbus Ohio USA

**Keywords:** balance billing regulations, emergency ground ambulance, health care prices, state insurance policies

## Abstract

**Objective:**

To examine the effects of New York's surprise billing regulations on price changes by emergency ground ambulance service providers.

**Study Design:**

We exploited a natural experiment using a difference‐in‐differences design with randomization inference (RI) to examine the effects of New York state regulations on the prices of emergency ground ambulances, analyzing 2012–2019 commercial claims data. In March 2015, New York implemented a law protecting patients from surprise out‐of‐network (OON) balance bills, including ground ambulance services. New York's policy tied OON ground ambulance reimbursements to usual, customary, and reasonable rates that typically reflect charges. The control group consisted of similar states that never enacted surprise billing laws. Although self‐funded plans are exempted from state laws, we also examined for spillover effects on self‐funded plans.

**Data Source and Analytic Sample:**

Multi‐payer national commercial plan claims data were used. The study sample was selected by isolating claims involving an emergency ground ambulance activation code.

**Principal Findings:**

The event study findings indicated that New York's law led to a continuous increase in prices, relative to controls. The law's implementation was associated with an overall emergency ground ambulance price increase of 13 percentage points (RI *p*‐value: 0.07). We observed a 21‐percentage‐point increase in in‐network prices (RI *p*‐value: 0.07) and a 19‐percentage‐point increase in OON prices (RI *p*‐value: 0.07), relative to controls, for fully insured health plans. Similar changes in overall prices and in in‐network prices were observed in self‐insured plans. Last, our study did not find statistically significant evidence of changes in out‐of‐pocket cost‐sharing amounts under New York's regulation.

**Conclusions:**

Balance billing laws based on charges can lead to price increases for emergency ground ambulance services. Legislation intended to inoculate patients from these surprise billings for ground ambulance transportation may have unintended consequences for costs of care.

**What is known on this topic:**

- Emergency ground ambulances are a major source of surprise billing.
- The federal No Surprises Act of 2020 excluded emergency ground ambulance services.
- Some states have regulations that prohibit out‐of‐network balance bills for ground ambulance services.

**What this study adds:**

- The study provides the first empirical evidence on the potential impacts of regulations on price changes among emergency ground ambulance providers.
- The study offers evidence of state policies' effects on fully insured plans and potential spillover effects on self‐funded plans.
- Experiences from New York's state ambulance out‐of‐network billing regulation indicate that tying reimbursement policies to charges may have the unintended consequences of increasing health care costs.

## 1 BACKGROUND

Managed care plans contract with health care providers who accept discounted pricing in exchange for in‐network participation. However, health plan members can encounter out‐of‐network (OON) providers unexpectedly, leaving them vulnerable to sizeable balance bills from these providers. This occurs frequently—over a 2‐year period, approximately 20% of insured adults reported a treatment episode with a potential “surprise” medical bill from an OON provider.^1^, ^2^ Moreover, patients typically face much steeper coinsurance requirements and separate or higher deductibles when using OON services.^3^

The frequency and size of surprise bills have generated consumer complaints and Congressional hearings. These concerns led to the passage of the federal No Surprises Act (NSA) in 2020, which prohibited OON balance bills in most situations when insured patients inadvertently received care from an OON provider or facility.^4^ The NSA was implemented in January 2022. It protected patients from surprise OON bills for most emergent and many non‐emergent clinical services, but ground ambulance services were excluded.^5^ Ground ambulance providers, which transport about 3 million privately insured patients to emergency rooms each year,^6^ are a large source of surprise bills.^7^ Recent data reveal that 79% of emergency ground ambulance episodes are OON.^8^ Thus, OON ground ambulance transports during an emergency can result in unexpected financial burdens for patients.

During a medical emergency, dispatchers send the closest ambulance and paramedics to provide medically necessary services and transit to an emergency room. This creates an especially egregious vulnerability for patients with precious little time or information to consider network participation as a prerequisite for choosing an ambulance provider. Thus, patients facing a medical emergency conventionally are unable to specify an in‐network ground ambulance provider. Moreover, incentives for network participation by ambulance providers are low. In contrast to other providers (e.g., physicians) and facilities (e.g., hospitals), ground ambulance providers are typically unaffected by OON status and transportation volumes are often sustained. This situation represents a classic market failure.

Because OON ground ambulance rides are not rare, this deficiency in the breadth of the NSA's coverage has meaningful consequences to consumers. In fact, recent data revealed that up to 8% of medical debt originates from OON ground ambulance surprise billings alone.^9^ Moreover, payments to ground ambulances are factored into consumers' health plan premiums. Thus, price increases may lead to an increase in insurance premiums. Because of the exclusion of ground ambulance coverage from the NSA, some states have enacted laws of their own to regulate surprise billings from ambulance providers.^10^ Amid growing concerns from consumers and policymakers, the NSA established the Advisory Committee on Ground Ambulance and Patient Billing, chartered to advise the Secretary of Health and Human Services, the Secretary of Labor, and the Secretary of the Treasury to make recommendations that protect consumers from balance billing with ground ambulance services. In November 2023, this federal committee recommended expansion of the NSA to include ground ambulance services and recommended that the payment amount comply with existing state balance billing laws, or in cases where the ground ambulance balance billing regulation is absent, request local government agencies to negotiate with insurers.^11^ Thus, the findings from our study of the effects of state‐level legislation are especially relevant for consumers, policymakers, and other stakeholders.

While many states have implemented regulations to address surprise billing issues, most have exempted ground ambulance providers. Nonetheless, several states have regulations that prohibit OON balance bills for ground ambulance services and specify that consumers are only responsible for in‐network cost‐sharing.^10^ In these states, providers are prohibited from balance billing and patients are shielded from out‐of‐pocket costs higher than the normal in‐network cost‐sharing requirements. The potential impacts of regulations on the prices of emergency ground ambulance providers are unknown. Therefore, experiences from recent state ambulance OON billing protections can be leveraged to answer important questions regarding the effects of recent state surprise billing laws on ground ambulance prices.

Since the Employee Retirement Income Security Act covers most self‐funded health plans, state laws on surprise billing are limited to state‐regulated “fully insured” plans. Nonetheless, health insurers typically offer self‐funded plans with the same provider networks and contracts. Thus, it is possible that the state laws that protect consumers in fully‐insured plans may also impact provider reimbursement for self‐funded plans, a so‐called “spillover effect.” Our analysis considers these effects as well.

Specifically, this study examines the effects of New York's state ground ambulance balance billing law that was implemented during the study time period. In March 2014, New York passed an “Emergency Medical Services and Surprise Bills Law.”^12^ On March 15, 2015, the law went into effect, protecting patients from surprise OON balance bills for many services, including ground ambulance services delivered by public and private owners. In the balance billing regulation, New York adopted an arbitration process and applied usual, customary, and reasonable (UCR) rates, typically based on a charge percentile, as the OON standard. According to the New York State's law on surprise billing, if there is a dispute between provider and insurer in a surprise billing case, including ground ambulance providers, both parties can go through an arbitration process.^13^ In practice, the law has advised that payments for OON ambulance providers should be based on the “usual and customary charge, which shall not be excessive or unreasonable.”^14^ Therefore, New York's payment rules could result in payments for OON ground ambulance services similar to established charges. New York's policy in establishing OON payment reconciliation allows us to estimate how rules for setting OON payment rates based on charges could affect ground ambulance prices. We further examine the question of whether there were “spillover” effects of the law designed for fully insured plans on self‐funded plans. In this study, “ground ambulance providers” includes all ground ambulance entities that provided services that were captured by the commercial dataset.

## 2 METHODS

### 2.1 Data

This study used 2012–2019 national multi‐payer claims data from the Health Care Cost Institute's 2.0 Commercial Claims Research Dataset, which covers one‐third of the employer‐sponsored insurance population in the United States. The study interval represented the latest data before the COVID‐19 pandemic disrupted health care activities. The data included detailed procedure information, insurer's allowed amount, patient cost‐sharing, dates of service, health plan type (e.g., HMO and PPO), patient residence state and five‐digit zip code, and the provider's encrypted provider identifier. A network status indicator allowed identification of in‐ and out‐of‐network claims. The data distinguished individuals enrolled in fully insured plans from those in self‐funded plans. However, the data did not contain charges, so we could not explore the difference between the claim's allowed amount and charge under New York's payment rule.

We merged the claims data with Medicare ambulance fee schedule files (for 2012–2019) from the Centers for Medicare and Medicaid Services, sorted by procedure code and zip code (using the Zip Code to Carrier Locality File provided by Centers for Medicare and Medicaid Services).^15^ Data on state balance billing laws that regulated ground ambulance services were collected from public reports, the state's legislative websites, and legal research databases.^16^

### 2.2 Identifying ground ambulance services

The study sample was selected by isolating claims involving an emergency ground ambulance activation code. Thus, we included claims with a Current Procedural Terminology code of A0427, A0429, or A0433. Most ground ambulance claims comprise multiple observations representing separate services (e.g., activation, mileage, and supplies). We included the reimbursements for activation, mileage, and other services associated with each claim. For mileage, this included A0021 (out‐of‐state mileage), A0380 (basic mileage), A0390 (advanced mileage), A0420 (waiting time), and A0425 (ground mileage). For supplies, this included A0382 (basic routine), A0384 (basic defibrillation), A0392 (advanced defibrillation), A0394 (advanced IV drugs), A0396 (advanced intubation), A0398 (advanced routine), and A0422 (oxygen). We also included A0420 (extra ambulance attendant), A0888 (non‐covered ambulance mileage), and A0999 (patient refused transport). We excluded claims from indemnity plans, claims for patients aged 65 and over, and secondary claims. We also excluded claims that were unlikely to be commercial managed care claims and claims with likely miscoded information or other problems. Inter‐facility transfer Current Procedural Terminology codes were not included because New York's regulation exempted these transfers. All claims that involved air ambulances were also excluded.

### 2.3 Outcome measures

We analyzed total prices, OON prices, and in‐network prices for emergency ground ambulance services. While the laws only directly regulate payments for OON ambulance providers, they may also affect in‐network prices by changing the outside options of the ambulance providers in their negotiations with private health plans. We measured the price for each claim as the total allowed amount, including both the insurer payment and the patient's cost‐sharing.

Because surprise billing laws could alter ambulance provider incentives for network participation, an analysis of in‐network or OON reimbursements could indicate a change in the composition of claims, even if the underlying prices have not changed. For example, providers with low OON reimbursement may reconsider network participation after the law was implemented. Thus, the mean OON reimbursement would increase, even if OON reimbursement levels remained the same. This is because of the removal of low‐reimbursement ambulance providers from the OON distribution. Therefore, the key outcome measure for our study was the total price, agnostic to network status.

#### 2.3.1 Measuring prices

To standardize the pricing and allow comparison across geographic areas, the ground ambulance prices were converted to a multiple of Medicare reimbursement for an identical claim. For example, a price of 1.5 meant that the reimbursement was 50% more than Medicare would have paid for the same ambulance claim. At the claim level, we first followed Medicare's formula to calculate the allowed amount by Medicare for the same service, considering the ambulance pickup location.^15^ We calculated the ground ambulance payments as a function of base rate, RVU, and mileage rates. The calculations differed by rural, super‐rural, and urban locations.

1. Urban ground adjusted base rates—(RVU*(0.3 + (0.7*GPCI)))*BASE RATE* 1.02.
2. Urban ground mileage rates—BASE RATE*1.02.
3. Rural ground adjusted base rates—(RVU*(0.3 + (0.7*GPCI)))*BASE RATE* 1.03.
4. Rural ground mileage rates—BASE RATE*1.03.

Medicare provides per‐transport reimbursements that vary by levels of service and account for the distance between ambulance pickup and drop‐off locations, with adjustments for rural pickups. Because Medicare reimbursement adjusts for differences in costs by pickup location via the geographic practice cost index and makes additional adjustments based on acuity and rural locations of service, our price measure implicitly controlled for these factors. In general, the Medicare reimbursement formula includes a base rate and an additional per‐mile rate. For transports from rural areas, Medicare increases both the base and mileage rates.^15^ Following Medicare's rule, we applied the special 1–17 rural mileage rate to the first 17 miles for rural activation claims. For the “super‐rural bonus” bonus payment, we multiplied rural ground ambulance transport service payment rate by 0.226.^15^

The actual pickup location was not included in the data. Following prior studies,^17^ we assigned the pickup location using the claim modifier, if present. For claims with modifier Residence to Hospital, the patient residence's zip code was used as the proxy for the pickup location. For claims a modifier that begins with H (except for HH), such as Hospital to Residence (HR) or claims with the modifier Scene of Accident to Hospital (SH), the hospital zip code, if present in the data, was used for the pickup location. For all other claims, we assume the patient's residence zip code is the pickup location. We were able to assign the point‐of‐pickup for 71% of the claims. As a robustness check, instead of assigning all non‐HR/SH claims to the patient's zip code, we only assigned those with a known modifier beginning with R (indicating residence) to the patient's zip code.

### 2.4 Research design

This study used a quasi‐experimental, difference‐in‐differences design with New York considered as the treated state. New York's law became effective on March 15, 2015, so the second quarter of 2015 was designated as the first quarter of treatment. To estimate the counterfactual New York outcomes had the laws not existed, we used a control group of states that never had ambulance balance billing regulations but had at least 100 OON claims in each quarter, like New York did.

### 2.5 Analyses

We estimated the quarterly treatment effects as well as the average treatment effects for New York's policy. Specifically, we estimated a two‐way fixed effects model with dynamic quarter‐from‐treatment indicators:

(1) $$Y_{\mathrm{ijt}}=\alpha_{j}+\delta_{t}+\sum_{l\in L}{\mu_{l}D}_{\mathrm{jt}}^{l}+\varepsilon_{\mathrm{ijt}}$$

where $Y_{\mathrm{ijt}}$ is the outcome of interest for claim *i* from state *j* in quarter *t*, $\alpha_{j}$ is a state‐specific effect, $\delta_{t}$ is a quarter‐specific effect, $D_{\mathrm{jt}}^{l}$ is an indicator that equals one when state *j* is *l* quarters from treatment, and $\varepsilon_{\mathrm{ijt}}$ is the idiosyncratic error.

To determine the average treatment effect of the New York law, we estimated

(2) $$Y_{\mathrm{ijt}}=\alpha_{j}+\delta_{t}+\mu D_{\mathrm{jt}}^{l\geq 0}+\varepsilon_{\mathrm{ijt}}$$

where $Y_{\mathrm{ijt}}$ is the outcome of interest for state *j* and claim *i* in quarter *t*, $\alpha_{j}$ is a state‐specific effect, $\delta_{t}$ is a quarter‐specific effect, $D_{\mathrm{jt}}^{l\geq 0}$ is an indicator that equals one for all claims in the treated state and all quarters after treatment, and $\varepsilon_{\mathrm{ijt}}$ is the idiosyncratic error.

Because there was only one treated state at the policy level for each analysis, conventional clustered standard errors may be biased downward. Therefore, inference for the average treatment effects was evaluated using randomization inference (RI), which is appropriate for estimating the significance level of treatment effects when there are few treated subjects.^18^, ^19^ With RI, placebo effects are estimated for each state in the control group as if it were the treated state. The *p*‐value is calculated as the proportion of placebo effects that exceeded the treated state's effect in absolute value. For example, a *p*‐value of 0.15 with an estimated effect of 0.25 indicates that, by chance, 15% of all random assignments produced an estimate of 25% or more, even in the absence of treatment. Given our conservative approach with RI, a *p*‐value of 0.10 was used to establish statistical significance.^19^ The analyses were conducted at the claims level.

### 2.6 Synthetic control analyses

The treatment effects estimated in Equations (1) and (2) rely on the parallel trend assumption that the counterfactual posttreatment outcomes of the treated state, had it not been treated, would have the same trend as outcomes of the control states after treatment. Although this assumption cannot be tested because the counterfactual outcomes are unobservable, it is commonly checked by comparing the pretreatment trends of the treated and control subjects. Because these pretreatment trends were not identical for several measures, we estimated Equation (2) with synthetic controls, which were weighted averages of the control states where the weights were selected to match each treated state with the synthetic control state in the pretreatment period, based on price and predictors of price. As predictors of price, we used the state's health expenditures per capita, the share of privately insured members in self‐funded health plans, and the Herfindahl–Hirschman Index (HHI) of concentration for the state's private health insurance market. We used the large group HHI for the self‐funded analysis and the small group HHI for the fully insured analysis. The state characteristics for each year from 2012 to 2019 were taken from Kaiser State Health Facts.^20^ For the synthetic control analysis, RI was conducted using the distribution of placebo ratios of the posttreatment root mean squared error to the pretreatment root mean squared error, to simultaneously capture the size of treatment effects relative to the placebo distribution and the quality of the pretreatment match between New York and its synthetic control.

### 2.7 Robustness analyses

We performed a series of robustness analyses, reported in the Appendices. First, we conducted robustness checks by using only claims where modifiers could be determined (Residence to Hospital, HR, and SH) which represents 52% of the claims, and the results are presented in Appendix A. We also conducted analyses using conventional clustered standard errors, and the statistical significance based on these *p*‐values is reported in Appendix B. We further conducted analyses with an alternative price measure: using a generalized linear regression (log link) to case mix‐adjust the allowed amount using quarter, sex, age band, health plan type (e.g., PPO and HMO), and the level of emergency service (i.e., basic, advanced level 1, and advanced level 2). Findings are reported in Appendix B. Last, we conducted analyses using the raw, unadjusted allowed amounts as prices (Appendix B).

## 3 RESULTS

As shown in Table 1, emergency ground ambulance claims in New York had a mean price ratio relative to Medicare of 1.34 for fully insured plans, implying that commercial ambulance claims were reimbursed 34% higher than Medicare on average. The prices were an average of 1.33 for OON claims and 1.33 for total prices. On average the allowed payment was about $1000, and patients paid an average of $232 from out‐of‐pocket cost‐sharing. The prices and allowed payments for self‐funded plans were similar to fully insured plans, but patients with self‐insured plans paid less for cost‐sharing on average. About 52% of the claims were in‐network in New York's fully insured plans.

**TABLE 1:** Summary statistics.

Fully insured	Fully insured	Self‐insured
Standardized in‐network price relative to Medicare price	1.34 (# of claims: 48,083)	1.27 (# of claims: 58,755)
Standardized OON price relative to Medicare price	1.33 (# of claims: 42,317)	1.07 (# of claims: 66,280)
Standardized total price relative to Medicare price	1.33 (# of claims: 90,400)	1.16 (# of claims: 125,035)
Total allowed reimbursements in dollars	$999.51 (# of claims: 134,229)	$911.23 (# of claims: 174,457)
Total reimbursed out‐of‐pocket price in dollars	$231.81 (# of claims: 134,229)	$146.87 (# of claims: 174,457)
Proportion of claims billed in‐network	51.81% (# of claims: 134,229)	45.58% (# of claims: 174,457)

Abbreviation: OON, out‐of‐network.

**FIGURE 1:**
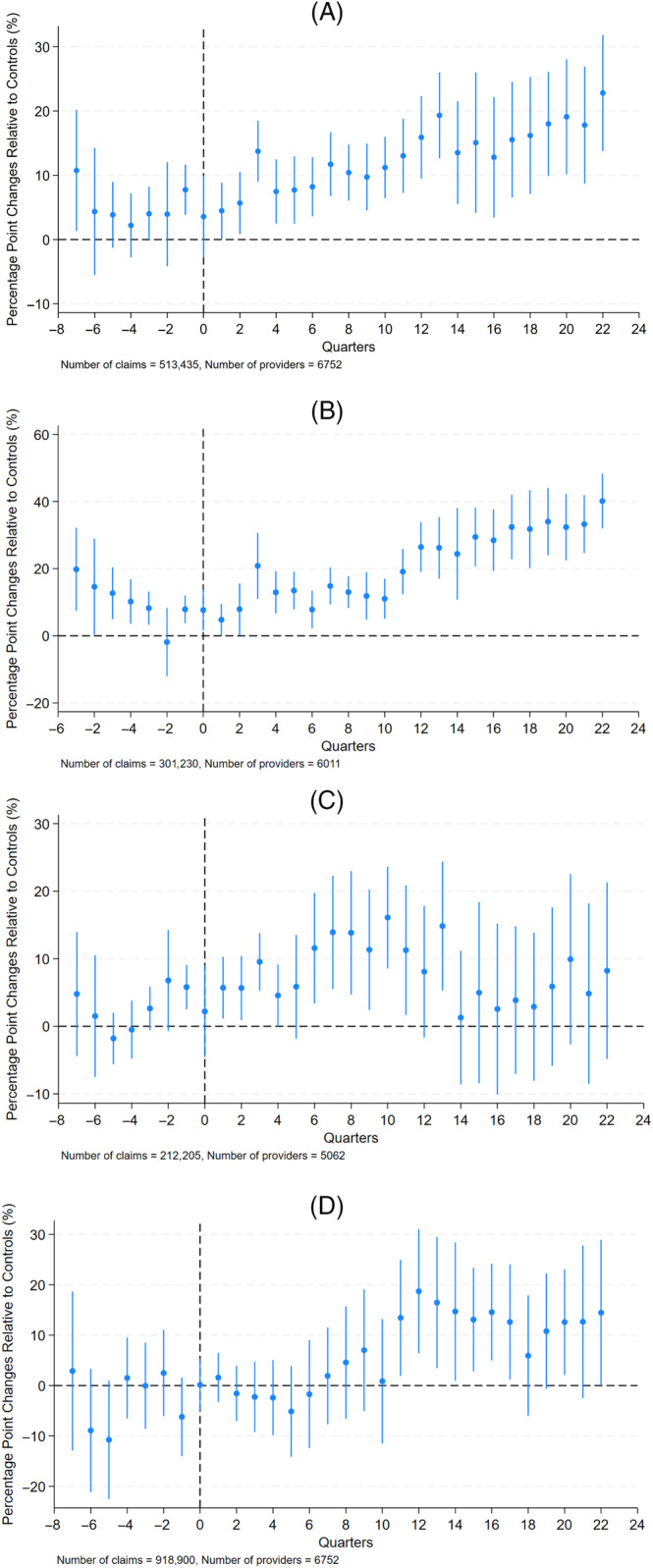
Percentage‐point changes in New York prices relative to controls, fully insured. (A) Total price. (B) In‐network price. (C) Out‐of‐network price. (D) Out‐of‐pocket cost‐sharing payments.

The event study graphs showing prices for fully insured plans are exhibited in Figure 1. The parallel trends assumption was met for the total price in most quarters before the second quarter of 2015, when the regulation went into effect (Figure 1A). Overall, relative to the controls, the New York state balance billing regulation led to a continuous increase in total prices for emergency ground ambulances. Increases were observed for in‐network and OON prices (Figure 1B,C), but both experienced price changes that started before the policy went into effect, consistent with the fact that the law was passed a year ahead of policy implementation. Further, differences between New York's OON price and control states disappeared after the initial few years following the legislation. As shown in Figure 1D, the parallel trends assumption was met for out‐of‐pocket cost‐sharing payments, and there were no significant differences between New York and the control states after the policy, except for a temporary increase in OOP payments.

**FIGURE 2:**
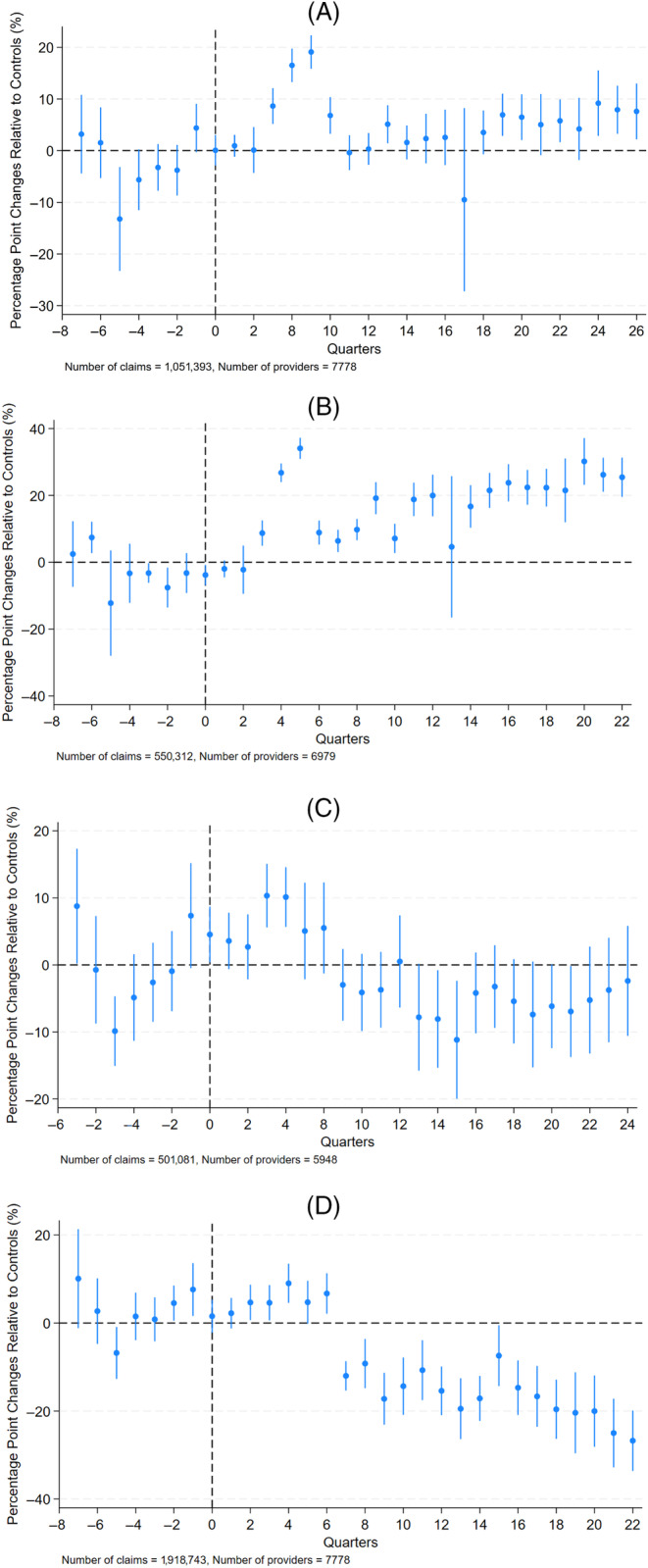
Percentage‐point changes in New York prices relative to controls, self‐insured plans. (A) Total prices, self‐insured plans. (B) In‐Network prices, self‐insured plans. (C) Out‐of‐network price, self‐insured plans. (D) Out‐of‐pocket cost‐sharing payments, self‐insured plans.

The event study graphs for self‐funded plans in New York are given in Figure 2. In general, the parallel trends assumption was not satisfied for the analysis of self‐funded plans. The self‐funded plans saw some increases in total price shortly after the state regulation, relative to the controls, but these changes were not sustained over time. The temporary increases were largely driven by in‐network price changes.

The average treatment effects for prices under New York's regulations are presented in Table 2. Findings from the average treatment analyses and synthetic control analyses were consistent and both are displayed. Because the parallel trend assumption was not always met, we focus more on the findings based on synthetic control analyses. In fully insured plans, the total price had a statistically significant increase of 13.34 percentage points after the policy, relative to the controls. The in‐network prices increased by 21.34 percentage points, while the OON prices of emergent ground ambulances in fully insured plans increased by 19.46 percentage points, both statistically significant changes, relative to control states under New York's balance billing regulation.

**TABLE 2:** Average treatment effects of New York's regulation on prices.

Outcome measures	Fully insured			Self‐insured		
	Total price	In‐network price	OON price	Total price	In‐network price	OON price
Marginal effects	8.19%	9.71%	8.12%	5.46%	13.64%*	−1.18%
# of providers	6752	6011	5062	7778	6979	5948
# of claims	664,568	413,002	251,566	1,452,301	844,406	607,895

	Total price (synthetic control)	In‐network price (synthetic control)	OON price (synthetic control)	Total price (synthetic control)	In‐network price (synthetic control)	OON price (synthetic control)
Marginal effects	13.34%*	21.34%*	19.46%*	9.45%	17.99%***	−4.97%
# of providers	7026	6251	5228	8074	7256	6139
# of claims	728,083	455,877	272,206	1,559,754	922,882	636,872

Abbreviation: OON, out‐of‐network.

*p < 0.1, **p < 0.05, ***p < 0.01.

The price changes for self‐funded plans generally followed the trend of those for fully insured plans. We observed a statistically nonsignificant increase in total price by 9.45 percentage points. We also found a statistically significant increase of in‐network prices by 17.99 percentage points, along with a small, statistically insignificant decrease in OON prices by 4.97 percentage points, relative to the controls.

Results in Table 3 indicate that fully insured patients had 12.38 percentage‐point higher out‐of‐pocket cost‐sharing payments relative to the controls, though the difference was not statistically significant. In contrast, the cost‐sharing amount for patients in self‐insured plans decreased by 6.40 percentage points (also not statistically significant) after the policy implementation.

**TABLE 3:** Average treatment effects of New York's regulation on out‐of‐pocket costs for cost‐sharing requirements.

Outcome measures	Fully insured		Self‐insured	
	Out‐of‐pocket price	Out‐of‐pocket price (synthetic control)	Out‐of‐pocket price	Out‐of‐pocket price (synthetic control)
Marginal effects	9.94%	12.38%	−11.90	−6.40%
# of providers	6752	7026	7778	8074
# of claims	918,900	1,038,082	1,918,743	2,103,811

*p < 0.1, **p < 0.05., ***p < 0.01.

Our analyses using the alternative pickup point assumption (Appendix A), the analyses using conventional clustered standard errors, those using GLM case control outcomes, and those using unadjusted raw allowed amounts (Appendix B) all confirmed the coefficient estimates of the main analyses. The only exception was price changes in OON prices for self‐insured plans, yet even there, all the analyses indicated very small and statistically insignificant changes relative to the controls.

## 4 DISCUSSION

In this quasi‐experimental study focusing on the association between New York's balance billing regulation and emergency ground ambulance prices, we found that the legislative remedy was associated with price increases for emergency ambulance services in fully insured plans. New York's OON reimbursement mechanism that considers UCR—typically based on 70%–80% of charges as the OON rate reference—resulted in overall price increases in OON payments for regulated plans. The OON price increases we observed could give providers more leverage in negotiations, leading to higher in‐network prices and total prices. These higher reimbursements would eventually translate to higher premiums for enrollees and employers. Our findings in self‐insured plans were largely consistent with the changes in fully insured plans. However, the increased price patterns relative to control states were not statistically significant except for an increase in the in‐network prices. These observations imply potential spillover effects on the self‐insured population, arising from New York's reimbursement policy that is tied to charges.

Ground ambulance transportation is a unique component of the health care system. In an emergency, patients and families are not able to choose providers when requesting ambulance services. Without a clear federal policy to prohibit balance billing after emergency ground ambulance transports, consumers may be subjected to unaffordable surprise bills for a service where patient choice is nearly impossible to exercise. The federal committee has recommended that the reimbursements for OON emergency ground ambulances be set based on the amount specified in a state balance billing law, or by state and local payment regulations. If no such rate setting exists, the payment is recommended to be benchmarked to Medicare payment rates. The federal committee rejected the use of arbitration as a resolution to determine payments.^5^

In the implementation of the NSA for other surprise billing situations, the federal government has allowed states to continue their payment rules for OON providers. So far, almost half of all states are using their existing state laws to determine payments from insurers in lieu of the federal system.^21^ Fourteen states enacted ground ambulance surprise billing laws, both before and after the passage of the NSA.^5^ If the NSA will grant similar state autonomy for potential federal rules concerning ground ambulance surprise billing, the state policy effects may be carried over, at least for fully insured plans. Policymakers should be aware that there are potential consequences from different reimbursement strategies, such as the UCR reimbursement setting used in New York, intended to mitigate surprise billing for ground ambulance services.

There are unique policy challenges to ensure that OON ground ambulance payments for surprise billings do not represent a disproportionate financial burden for patients, their families, or for providers themselves. Ground ambulance services are delivered by both public entities and private companies. In fact, fire departments provide more than 60% of emergency transports in the United States, and the rest are split between private equity, publicly traded companies, facilities (e.g., hospitals), and independent ground ambulance providers.^6^, ^17^ Medicare and Medicaid reimbursements, typically referenced by commercial payers, are based on the average Emergency Medical Service rates in each region. This furnishes incentives for local providers to set high transport fees.^22^ Meanwhile, tax revenues cover a substantial portion of ground ambulance services provided by public agencies, and some providers may not participate in any networks or bill insurance. The balance billing policies, which regulate reimbursements and billing behaviors, are typically administered by the Department of Insurance in each state. Yet, the ground ambulance providers can represent a variety of entities governed by other government branches. The unique provider ownership mix creates complex policy challenges to regulate ground ambulance OON billing practices.

Our study has some limitations. First, our estimates of OON prices are likely conservative. Because balance bills are sent by the provider to the consumer directly, we cannot observe the actual balance bills in our data. Second, some public providers may not bill any insurance. Thus, our study's findings cannot be generalized to all ground ambulance providers. Moreover, we did not have data on charges. Therefore, the comparison of reimbursements with charges was out of the scope of this study. Further, some unobserved market changes may have happened during our observation period that cannot be fully captured in a secondary data analysis. Lastly, while our analyses were built on a rigorous quasi‐experimental design, our findings may indicate association instead of causation.

Our study on the effects of New York's law on ground ambulance pricing is especially germane to evolving policies that seek to protect consumers from surprise medical bills from OON providers. Our findings raise the question of whether state regulations are effective approaches to address the market failure of surprise billing for ground ambulance services. Some state legislation intended to inoculate customers from surprise billings from ground ambulances may have unintended consequences and raise the overall costs of care as a result.

## Supporting information


**Appendix A.** Average treatment effects of New York's regulation on prices, alternative pick‐up location assignment
**Appendix B.** Average treatment effects of New York's regulation on outcomes.
