# Supplementary material for: The impacts of New York's balance billing regulation on ground ambulance pricing
Source: Health Serv Res. 2024 Oct 16;60(2):e14387. doi: 10.1111/1475-6773.14387 (PMC11911222; doi:10.1111/1475-6773.14387)
Supplement: Supplementary file 1 — Appendix A. Average treatment effects of New York's regulation on prices, alternative pick‐up location assignment Appendix B. Average treatment effects of New York's regulation on outcomes. [file HESR-60-0-s001.pdf]

APPENDIX A: Average treatment effects of New York's regulation on prices, alternative pick-up location assignment

	Fully insured			Self-insured		
Outcome measures	Total price	In-network price	Out-of-network price	Total price	In-network price	Out-of-network price
Marginal effects	8.47%**	12.87%	5.52%	7.16%***	20.18%***	−1.38%
# of providers	6752	6011	5062	7778	6979	5948
# of claims	513,435	301,230	212,205	1,051,393	550,312	501,081
	Total price (synthetic control)	In-network price (synthetic control)	OON price (synthetic control)	Total price (synthetic control)	In-network price (synthetic control)	OON price (synthetic control)
Marginal effects	4.43%	−4.01%	11.97%	10.77%	12.98%	−5.21%
# of providers	7026	6251	5228	8074	7256	6139
# of claims	555,952	326,467	229,485	1,115,883	591,538	524,345

Abbreviation: OON, out-of-network.
*$p < 0.1$, **$p < 0.05$, ***$p < 0.01$.

APPENDIX B: Average treatment effects of New York's regulation on outcomes

	Fully insured			Self-funded		
Outcome measures	Total price	In-network price	Out-of-network price	Total price	In-network price	Out-of-network price
Marginal effects from clustered standard errors	8.19%***	9.71%***	8.12%*	5.46%***	13.64%***	−1.18%
# of claims	6752	6011	5062	7778	6979	5948
# of providers	664,568	413,002	251,566	1,452,301	844,406	607,895
Marginal effects, GLM-adjusted price measures	7.02%*	14.00%***	2.71%	7.02%	13.21%*	1.82%
# of claims	6752	6011	5062	7778	6979	5948
# of providers	918,900	541,481	377,419	1,918,743	1,058,763	859,928
Marginal effects, raw allowed amounts as price measures	9.86%	17.12%*	7.05%	8.42%	17.96%***	1.34%
# of claims	6752	6011	5062	7778	6979	5948
# of providers	918,900	541,481	377,419	1,918,743	1,058,765	859,978

*$p < 0.1$, **$p < 0.05$, ***$p < 0.01$.
